# A lightweight convolutional neural network model with receptive field block for C-shaped root canal detection in mandibular second molars

**DOI:** 10.1038/s41598-022-20411-4

**Published:** 2022-10-17

**Authors:** Lijuan Zhang, Feng Xu, Ying Li, Huimin Zhang, Ziyi Xi, Jie Xiang, Bin Wang

**Affiliations:** 1 Department of Oral Medicine, Shanxi Provincial People’s Hospital, Taiyuan, China; 2 College of Information and Computer, Taiyuan University of Technology, No. 79, Yingze West Street, Taiyuan, 030024 Shanxi China

**Keywords:** Dentistry, Dental diseases

## Abstract

Rapid and accurate detection of a C-shaped root canal on mandibular second molars can assist dentists in diagnosis and treatment. Oral panoramic radiography is one of the most effective methods of determining the root canal of teeth. There are already some traditional methods based on deep learning to learn the characteristics of C-shaped root canal tooth images. However, previous studies have shown that the accuracy of detecting the C-shaped root canal still needs to be improved. And it is not suitable for implementing these network structures with limited hardware resources. In this paper, a new lightweight convolutional neural network is designed, which combined with receptive field block (RFB) for optimizing feature extraction. In order to optimize the hardware resource requirements of the model, a lightweight, multi-branch, convolutional neural network model was developed in this study. To improve the feature extraction ability of the model for C-shaped root canal tooth images, RFB has been merged with this model. RFB has achieved excellent results in target detection and classification. In the multiscale receptive field block, some small convolution kernels are used to replace the large convolution kernels, which allows the model to extract detailed features and reduce the computational complexity. Finally, the accuracy and area under receiver operating characteristics curve (AUC) values of C-shaped root canals on the image data of our mandibular second molars were 0.9838 and 0.996, respectively. The results show that the deep learning model proposed in this paper is more accurate and has lower computational complexity than many other similar studies. In addition, score-weighted class activation maps (Score-CAM) were generated to localize the internal structure that contributed to the predictions.

## Introduction

Root canal systems have many anatomical variations, some of which can significantly increase the difficulty of endodontic treatments^1^. Cooke et al.^2^ first explicitly proposed the existence of type C root canals in mandibular second molars^2^. Walker et al. found a high incidence of type C root canals in mandibular second molars of 52% in China^3^. The root canal orifice of type C was 180°. The C-shaped banded grooves with the reticular connection between the root canals form a structurally complex isthmus^4^. The root cross-section of the C-shaped root canal is “C” type because the tooth germ on the buccal, lingual side of the root is not or only incompletely fused during development, resulting in the formation of crown roots with longitudinal groove and deformation of the extruded root canal^5^. Therefore, it becomes a challenge for clinicians to treat C-shaped root canal teeth.

Panoramic radiography remains the standard imaging method during preoperative assessment of the tooth root canal^6^. It displays each dentition and jaws on a single film through a fast and efficient process^7^. Cone-beam CT (CBCT) is a common technique used by dentists to accurately assess root and root canal morphology. Nevertheless, CBCT has a larger radiation volume for patients than panoramic radiography. It is not suitable to just make a simple diagnosis for patients^8,9^. Therefore, direct identification of C-shaped tooth root and root canal morphology by panoramic images will be effective in helping clinical diagnosis.

However, the correct identification of C-shaped root canals requires many years of experience. The same physician’s judgment of tooth root canals by CT can be limited by time and fatigue. Radiology awareness was shown to reduce the performance of root canal classification detection in the interpretation of CT of teeth due to long working hours^10^. Accurate classification of the type of tooth root canal plays a crucial role in the clinical management of the patient as well as in the prognosis.

Deep learning^11,12^, a hot research direction in the field of machine learning, has made breakthrough progress in computer vision and other artificial intelligences in recent years. CNNs are the most commonly used techniques when processing images because of their ability to extract rich details from the input information^13^. They have been successfully for image-based automated diagnosis in various fields, including lesion detection or classification^14–19^ and medical image segmentation^20–23^. In the field of dentistry, studies are being conducted using CNNs on various topics: morphological classification of teeth^24^, classification of dental implant systems^25,26^, dental caries^27^, mandibular canal segmentation^28^, and segmentation of teeth^29^. However, in pursuit of a higher precision effect, most of these studies often ignore the computational and parametric sizes of the CNN models used. Neural networks are typically over-parameterized and there is significant redundancy for convolutional neural network models^30^. Such CNNs are not suitable for hardware resource-constrained environments^31^.

This study proposes a new lightweight CNN structure that is more suitable for C-shaped root canal detection. The proposed method contains a module that can simulate human visual perception, which improves accuracy while reducing the number of parameters and computational complexity. This design will greatly optimize the use of CNN models in resource-constrained environments.

## Material and methods

### Classification of the root canal of mandibular second molar teeth

**Figure 1:**
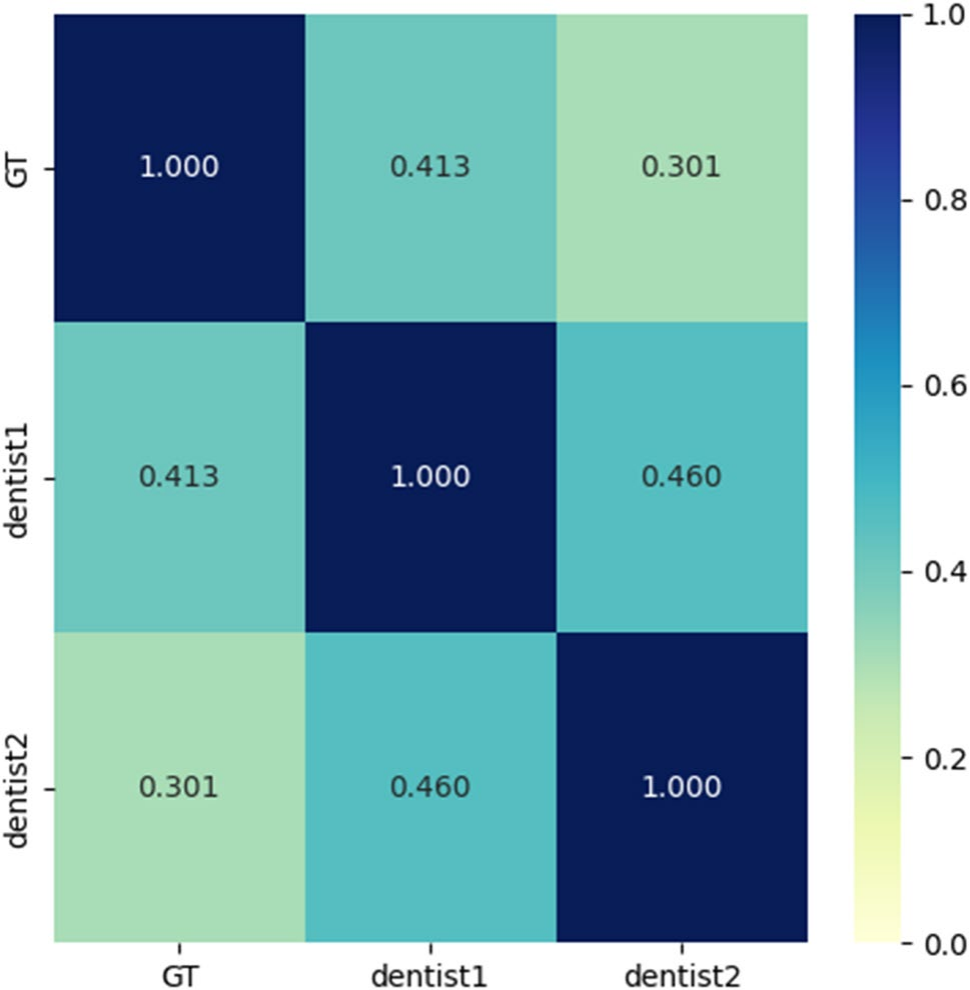
Evaluation of panoramic image datasets by two dentists and the Kappa test coefficient of the ground truth.

There are different variants of mandibular second molars, which mainly have two canals, three canals and C-shaped root canals^32^. In the clinical practice of root canal therapy, dentists use radiographs to assess the patient’s teeth and root canals, obtain relevant clinical information and predict the treatment difficulties. Nevertheless, dentists usually take much time and energy to judge the root canal type based on radiological images. They have difficulty determining the root canal type directly from panoramic images. In order to gain the dentists’ accuracy when assessing the mandibular second molar using only panoramic images, two dentists on the team classified the datasets blind to the labels. This work uses the Kappa^33^ test to calculate consistency with ground truth and its classification scores based on the classification scores of two dentists. Finally, these correlation values are constructed into a confusion matrix and the results are presented in Fig. 1. The coefficient of agreement between the two dentists and the ground truth were 0.301 and 0.413 respectively, and the coefficient of agreement between the two dentists was 0.46. It can be seen that dentists cannot well judge the root canal type of mandibular second molars from panoramic images.

### Dataset

This prospective work was conducted at the Department of Stomatology, Shanxi Provincial People’s Hospital. This research included panoramic image data from patients who received in-hospital dental care between August 2019 and November 2020 and obtained informed patient consent. Table 1 below describes the gender and age information of the patients included in this work.

**Table 1:** Baseline characteristics.

		Total (n = 384)
Age (year)	Median	27 (11–59)
	F	254
Sex	M	130
	%F	66.1%

**Figure 2:**
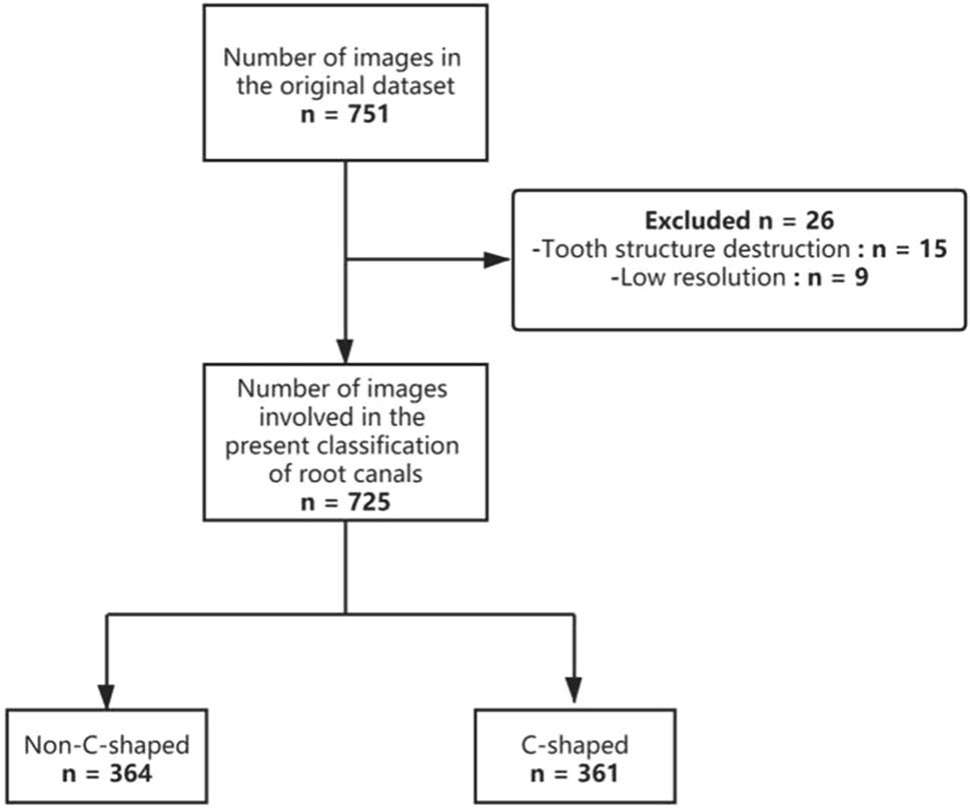
The image data flow chart defines how to decompose the data set.

The data set was marked by a radiologist with 5 years of professional experience and consisted of 384 panoramic images. The first step is the cutting stage. The area related to the mandibular second molar is selected by the manual cutting method and its size is adjusted to 90 × 90. The non-square graph is filled with zero. The second step is the cleaning stage, without the tooth with structure damage, low resolution and other unsatisfactory tooth images. After that, two team dentists conducted a second review of the data set and confirmed the availability of the basic facts. The final number of images: 361 cases of C-shaped root canals and 364 cases of non-C-shaped root canals. Figure 2 shows the whole process of data set production.

### The lightweight convolutional neural network with RFB

**Figure 3:**
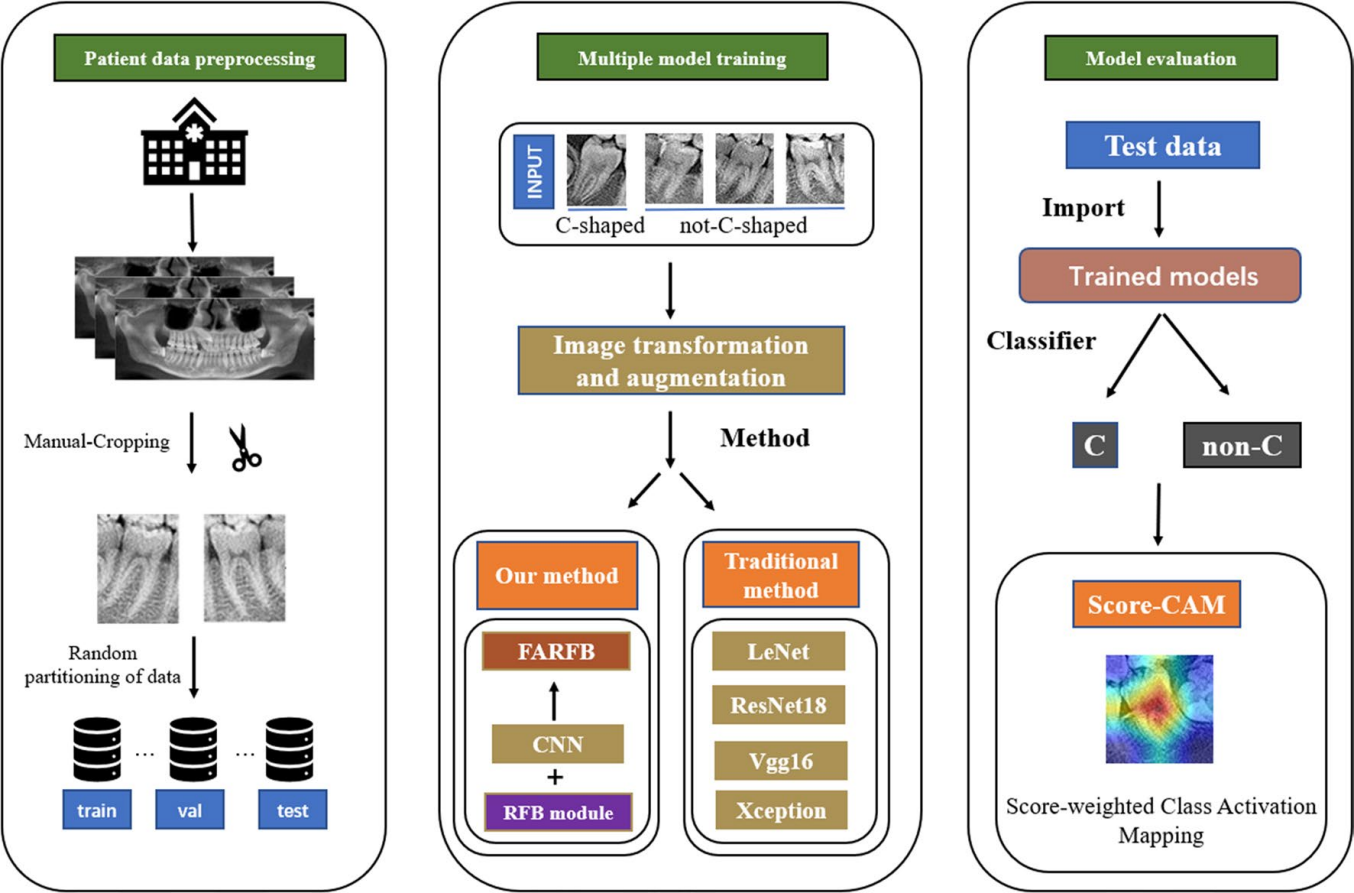
The workflow chart for classification of the tooth root canal. Cut out the mandibular second molar tooth image from the panoramic image produced from the original image data. The tooth image data obtained in the previous step was inputted into different network systems to classify and compare the results. The tooth image data gained in the last step was inputted to the proposed network combining increasing receptive field modules and classical network structure method. Finally, Score-CAM is used to visualize where the network focuses when making classification decisions.

In this paper, a new neural network structure is proposed to classify root canal types, as shown in the flowchart in Fig. 3. The crucial task in this work is the classification of C-shaped root canals and non-C-shaped root canals.

**Figure 4:**
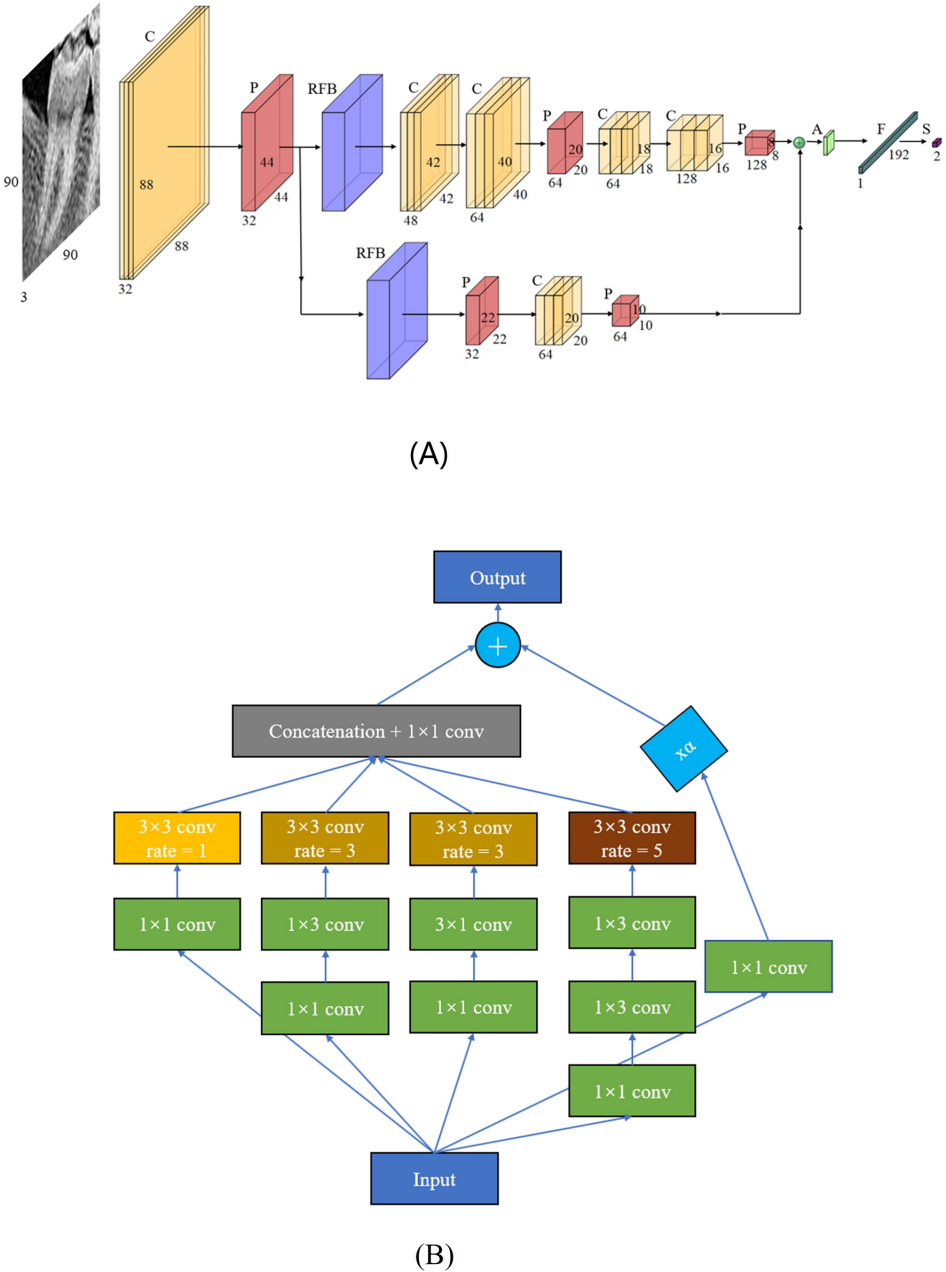
The network structure of FARFB (**A**). The capital letters A, C, F, P, S and S denote the Global Average Pooling, Convolutional, Full Connection, Pooling and SoftMax respectively. The values represent the number of channels, width and height of the feature maps. The composition structure of receptive field block (RFB) (**B**).

Considering the hardware constraints of the equipment and the time constraints of actual operation, this paper proposes a network FARFB that has been repeatedly debugged and modified and the network structure gives the model the best test accuracy. The network structure is shown in Fig. 4. Various network architectures and methods such as LeNet, Vgg16 and Xception were also tried in this document. The results of different network structures and methods on this data set are shown in Table 2. Experimental results show that the proposed method has better accuracy and lower resource consumption.

**Table 2:** Comparison of results and model indicators of fivefold cross-validation of FARFB and FANet.

Model	Accuracy	The number of parameters	The amount of calculation
FANet	0.9460	0.13 M	103.4 MFlops
FARFB	0.9838	0.18 M	139.5 MFlops

The network’s last layer is replaced by the SoftMax layer, which is used to classify several categories. After trying different configurations, this paper used 32 batches and an SGD optimizer with a momentum of 0.9 and a learning rate of 0.001. The function used to calculate the loss was the NLLLoss. This paper also used a 30-degree rotation range, horizontal flips and parallel shifts from height or width for data augmentation in the training set. This paper uses LeNet, Vgg16 and Xception to do the same calculations and keep the same parameters (except that the input image is 224 × 224 and 299 × 299 pixels). Finally, this paper chooses a combination of a lightweight convolutional neural network and RFB module as the best architecture for the current problem. Finally, to better understand the effect of the network, this paper can visually explain it and make better decisions on the model. This paper uses score-weighted class activation mapping (Score-CAM)^34^ to look at the areas where the network focuses when making decisions.

### Multi-scale receptive fields block

FARFB proposes to extract multi-scale receptive domain features to preserve the details of tooth images and better detect C-shaped root canals. Therefore, this work has to design a network with kernels of different sizes, such as 3 × 3, 5 × 5. To lightweight this method, this paper uses a small convolution kernel instead of a large one, which usually significantly increases the temporal complexity of the model. In this work, Receptive Field Block (RFB)^35^ was introduced into this network structure for the sake of lightweight and better classification. RFB is combined with a lightweight CNN model for extracting deep semantic features from the images. In particular, RFB uses a multi-branch convolution structure and different size convolution kernels correspond to different receptive fields, applies extended convolution layers^36^ to control their eccentricities and reshapes them to produce the final representation. In the proposed method, RFB is used to find the deep and rich details of tooth root canal images.

In FARFB, an RFB module is added to the backbone network, and a branch with an RFB module is added to obtain different levels of features and feature fusion is performed at the end of the network structure. The composition structure of RFB is shown in Fig. 4B. In RFB, in addition to using 3 × 3 convolution layer to replace the 5 × 5 convolution layer, 1 × 3 and 3 × 1 convolution layers are also used to replace the 3 × 3 convolution layer. The main purpose of this is to effectively reduce the amount of computation and parameters. In addition, replacing this structure enables RFB to have a richer semantic information learning ability. A corresponding dilated pooling layer follows each branch’s specific kernel-size convolution layer. Kernel size and dilated manipulation have similar positive correlations with RF size and eccentricity in the human visual cortex. Finally, the feature mappings of all branches are connected. This makes RFB precisely what this paper needs to extract multi-scale features and minimize time complexity simultaneously. The most important reason for using RFB is that it can extract very detailed features required for medical image classification.

### Training and evaluation

**Figure 5:**
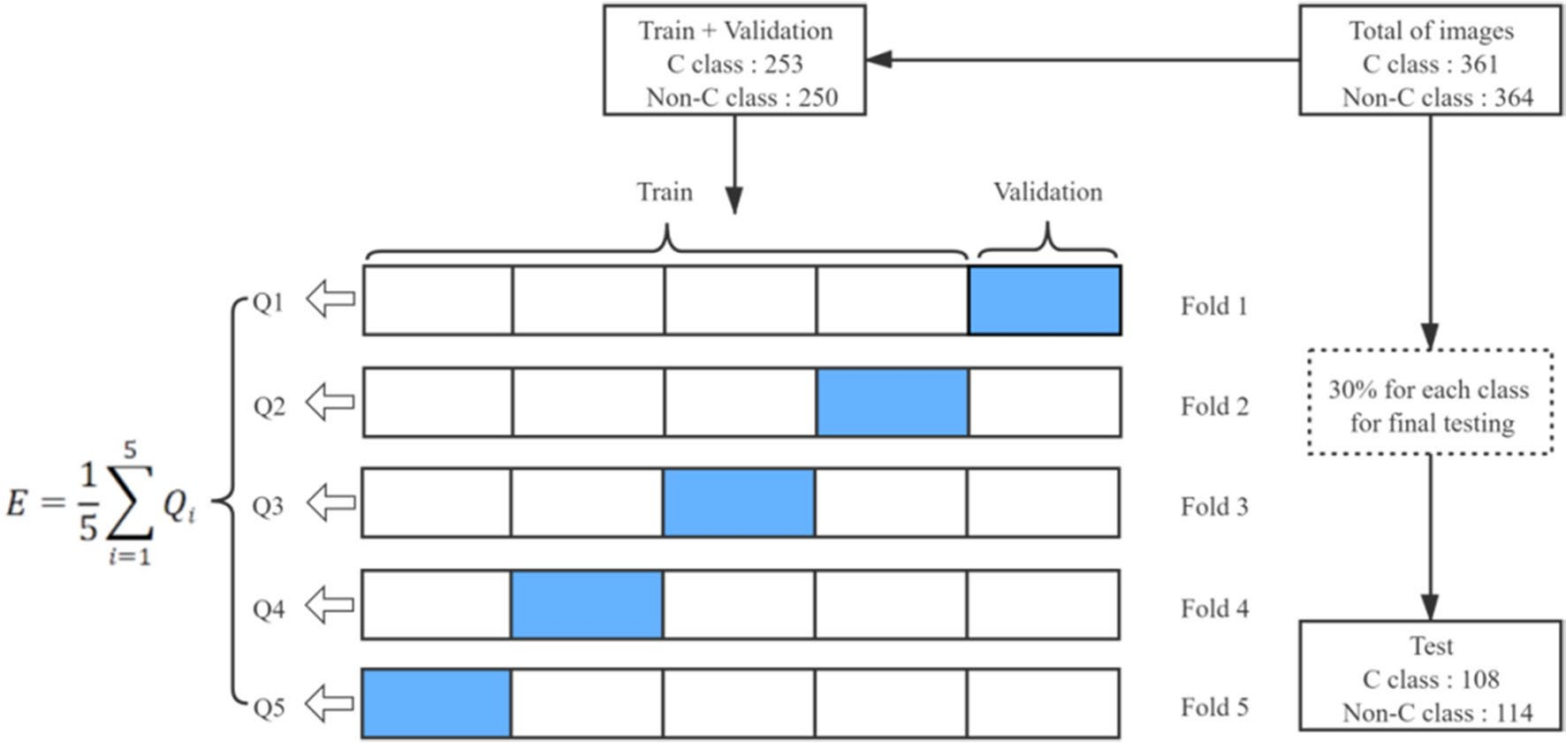
The details of the train set, validation set and test set composition.

From the initial data set, 30% of images of each category are tested separately. A data set composed of 114 images of non-C-shaped root canals and 108 C-shaped root canals was generated. Then, the remaining images (250 images of non-C-shaped root canals and 253 images of C-shaped root canals) were used for training. The partitioning of the data set is done using open-source random algorithms. This process is shown in Fig. 5.

This paper ran the model with 150 iterations and used a premature stop strategy with ten iterations. This paper used Pytorch, a well-regarded open-source framework for building deep learning networks, running on Ubuntu 18.04.2 LTS with NVIDIA GeForce GTX 2070. This paper calculated the accuracy of each network in judging C-shaped and non-C-shaped root canals and measured the performance of the CNN model using area under the receiver operating curve (AUC), precision, recall and F1-score^37^. Every value was averaged among the fivefolds. Finally, this paper compares the diagnostic performance of the proposed method with the diagnosis of dentists, where one dentist (experience > 5 years) and another dentist (experience > 8 years) predicted C-shaped canals from panoramic images. Dentists evaluated panoramic images of the mandibular second molars corresponding to all test sets. The accuracy, precision, recall and AUC were calculated and compared with the proposed method.

### Ethical approval

Research on human participants has been ethically reviewed and approved according to the requirements of the ethics committee of Taiyuan University of technology. According to national legislation and institutional requirements, this study has obtained written informed consent from the investigators.

## Result

### Performance of FARFB

Using the above FARFB model, this work achieved an average accuracy of 0.9838 for the C-shaped root canal classification of mandibular second molars (Table 2). At the same time, this paper also received other performance indices of the model, such as specificity, sensitivity and AUC of 0.9772, 0.9888 and 0.9960, respectively (Fig. 7). For the training results of the proposed method, this paper uses the fivefold cross-validation to get the average as this final result. With fivefold cross-validation, the accuracy of each fold of the model was 0.9775, 0.9865, 0.9820, 0.9820 and 0.9910, respectively. From the results, it can be seen that the proposed method has very excellent performance on each fold.

### Ablation study

In this work, the method was developed in combination with the RFB module. Therefore, in this ablation study, the RFB module was removed from the proposed method as the baseline method and named it FANet. This paper compares the FARFB, which only adds the RFB module to the network structure. In this work, the same dataset and hyperparameter settings were used for both methods. The results and network model indicators of the two methods on this dataset are shown in Table 2.

The results show that the FARFB method performs better. Although FANet has fewer parameters and fewer computation than FARFB, the results of FANet are not satisfactory. FARFB achieves more than 3% higher accuracy with slightly increased parameters and calculations than FANet. This result verifies the effectiveness of the proposed FARFB method.

### Comparison with traditional methods

**Figure 6:**
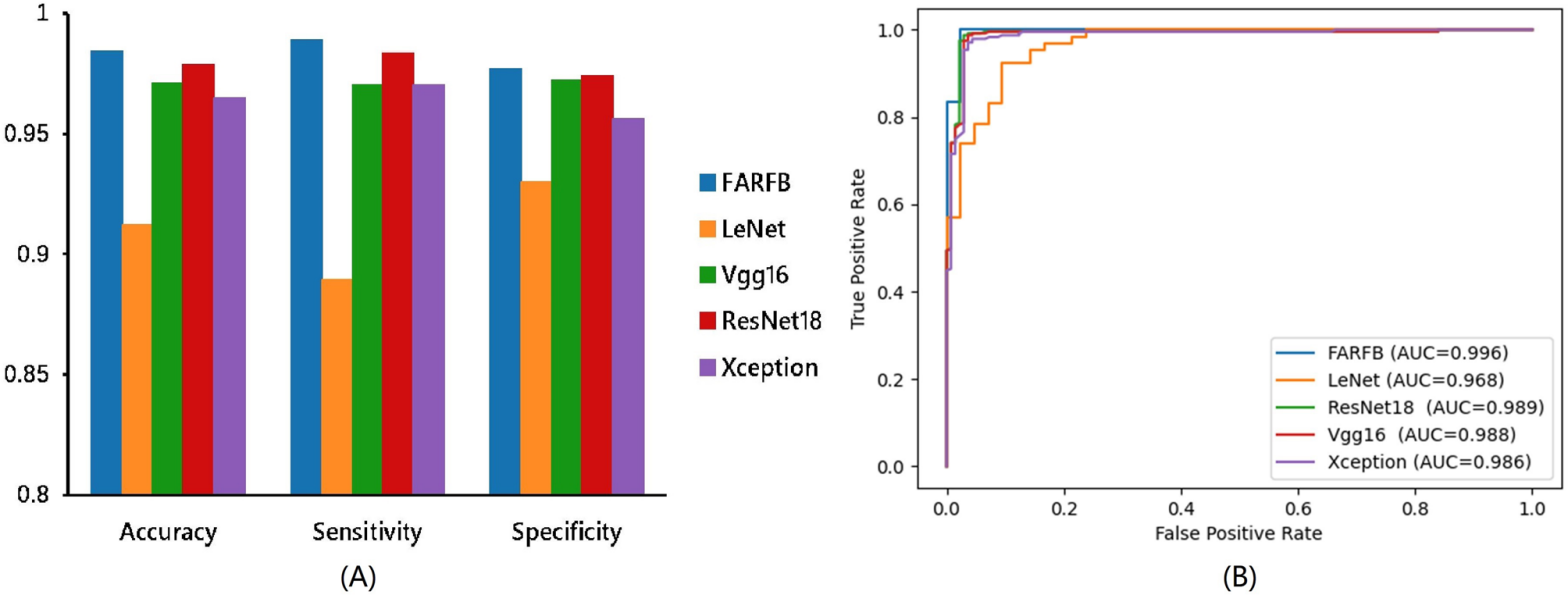
Comparison of the proposed method with traditional methods in accuracy, sensitivity, specificity (**A**). Receiver operating characteristic (ROC) curves for the proposed method and traditional methods (**B**).

In Fig. 6 and Table 3, this paper compares the proposed method with traditional methods in terms of accuracy, sensitivity, specificity and AUC. The accuracy, sensitivity, specificity and AUC of LeNet were 0.9117, 0.8893, 0.9298 and 0.9680, respectively. The accuracy, specificity, sensitivity and AUC of Vgg16 were 0.9712, 0.9880, 0.9704 and 0.9888, respectively. The accuracy, sensitivity, specificity, sensitivity and AUC of ResNet18 were 0.9784, 0.9737, 0.9834 and 0.9890, respectively. The accuracy, specificity, sensitivity and AUC of Xception were 0.9649, 0.9561, 0.9703 and 0.9860, respectively. From the above, it emerges from this paper that the proposed method shows a specific improvement in each performance index compared to the traditional methods.

**Table 3:** Accuracy comparison of LeNet, FARFB, Vgg16, ResNet18 and Xception in fivefold cross-validation training.

K-Fold	FARFB	LeNet	Vgg16	ResNet18	Xception
Fold1	0.9775	0.8964	0.9730	0.9730	0.9595
Fold2	0.9865	0.9234	0.9775	0.9820	0.9730
Fold3	0.9820	0.9144	0.9640	0.9775	0.9640
Fold4	0.9820	0.9054	0.9685	0.9730	0.9685
Fold5	0.9910	0.9189	0.9730	0.9865	0.9595
Average	**0.9838**	0.9117	0.9712	0.9784	0.9649
Standard deviation	± 0.0001	± 0.0001	± 0.0001	± 0.0001	± 0.0001

The best performance is shown in bold.

**Figure 7:**
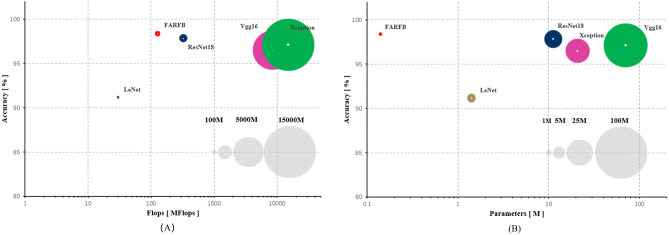
The graphical performance of each network model, the number of its parameters and calculations in C-shaped and non-C-shaped root canal classification tasks (the input image size of Vgg16 is 224 × 224. The input image size of Xception is 299 × 299). The gray circles are the standard display of the number of parameters or calculations at each level.

In addition, as shown in Fig. 7, the proposed method has a minimum number of parameters and a relatively small amount of calculation. The parameters of FARFB, LeNet, vgg16, resnet18 and Xception are 0.18, 1.41, 70.30, 11.20 and 20.80, respectively (Million). Their amount of calculation are 139.50, 29.91, 15,440.00, 324.79 and 8410.00, respectively (Million Floating-point Operations per Second, MFlops). The results show that the FARFB network has minimal parameters and amount of calculation, but extremely high accuracy. This is also the ideal result of the initial design of this network structure in this paper.

### Comparison with dentists and other studies

**Figure 8:**
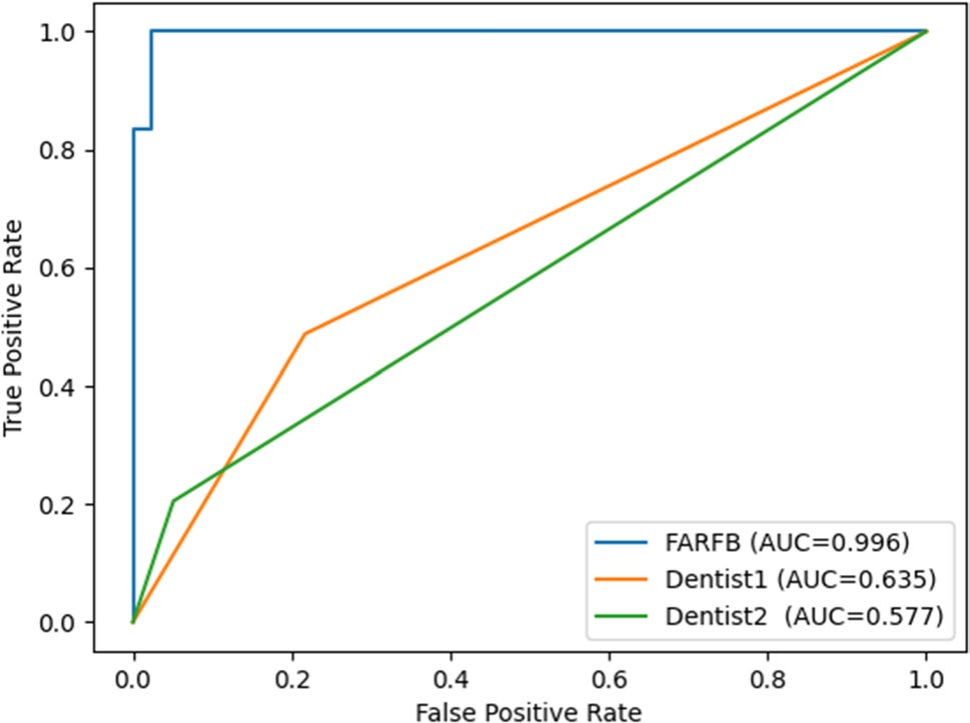
Receiver operating characteristic (ROC) curves for the proposed method and dentists.

It is usually a challenge for dentists to find C-shaped root canals directly through panoramic images. Table 4 and Fig. 8 show the performance indicators of the proposed method and two dentists in this C-shaped root canal classification task. It can be seen that the accuracy of the two dentists is 0.6352 and 0.5770, respectively, and the AUC are 0.635 and 0.577. This shows that the proposed method has better performance than two dentists.

**Table 4:** Comparison of diagnostic performance between the CNN model and the dentists.

	Accuracy (%)	Precision (%)	Recall (%)	F1-score (%)	AUC
FARFB	**98.38**	**97.81**	**98.88**	**98.34**	**0.996**
Dentist1	63.52	77.25	45.28	57.09	0.635
Dentist2	57.70	82.10	21.67	34.29	0.577
Jeon et al.^38^	95.10	95.90	92.70	94.27	0.982

The best performance is shown in bold.

Table 4 also shows that the proposed method performs much better than dentists and is also better than previous methods. Compared with the method of Jeon et al., the proposed method has a 3% higher accuracy on the test set and other performance metrics are also higher.

### Feature visualization of tooth images

**Figure 9:**
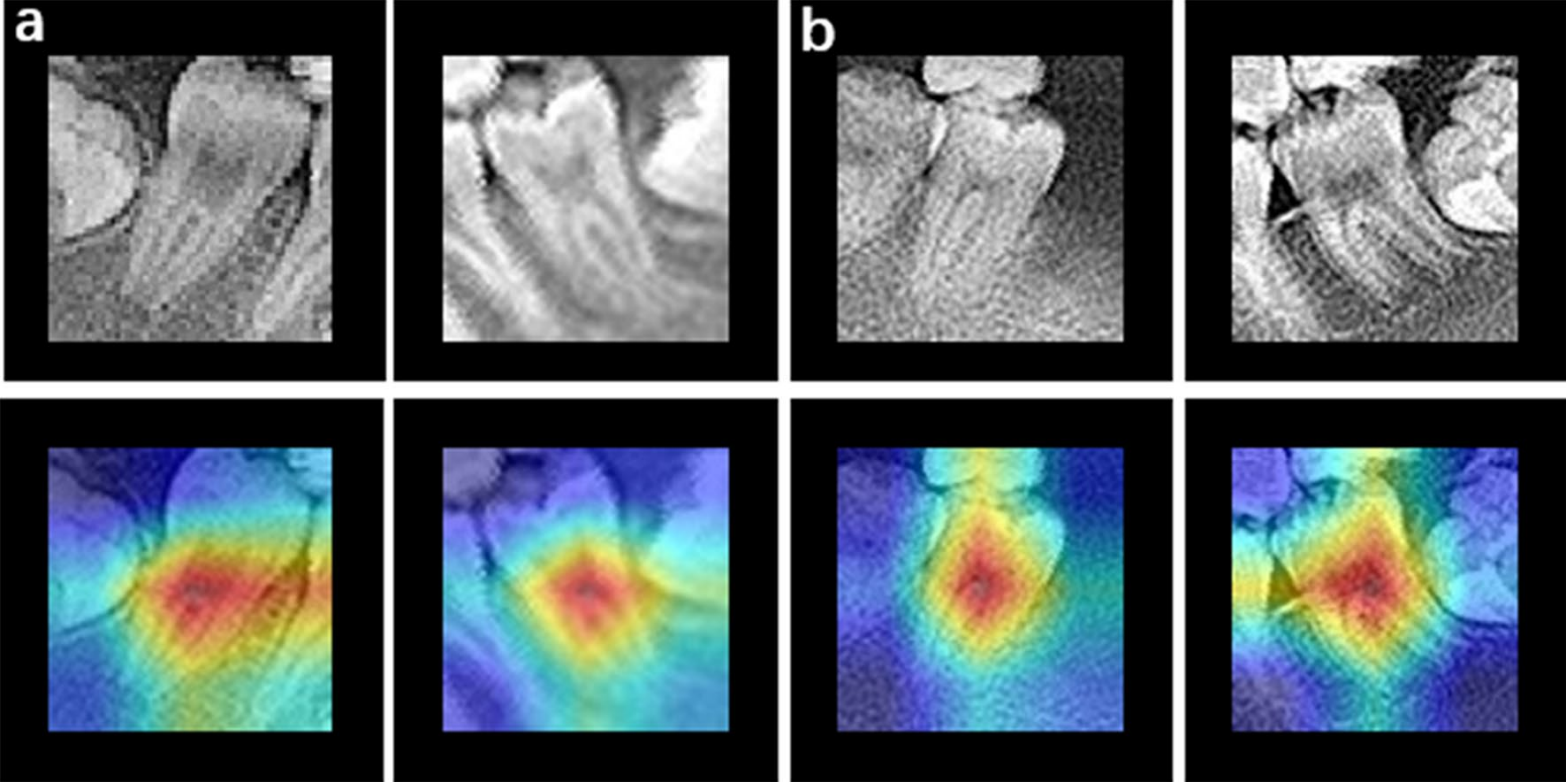
Score-CAM output for C-shaped and non-C-shaped type fractures classification. C-shaped root canal tooth images (**a**) and non-C-shaped root canal tooth images (**b**). Original images (top) and overlapped on the original image (bottom). The visualization certifies that the network is focusing on the correct area of the tooth.

Figure 3 shows images of C-shaped canals and non-C-shaped canals predicted using the proposed method and visualized using Score-CAM. The proposed method focuses on the feature of the intersection area of the root canal with the crown in the image and uses this feature to predict C-shaped root canals (Fig. 9a) correctly. The method focuses on the features of the area of the root canal bifurcation in the image and uses this feature to predict non-C-shaped root canals (Fig. 9b) correctly.

## Discussion

In this paper, a lightweight convolutional neural network model was proposed to predict C-shaped root canals in mandibular second molars by analyzing panoramic images. This paper selects several commonly used and highly representative methods in tooth image research for an experimental comparison and hopes to compare them to evaluate the effectiveness of the proposed model. Through several cross experiments, the network model proposed in this paper has the advantages of low resource consumption and high accuracy (Fig. 7). The accuracy of the proposed model in the classification of C-shaped root canals and non-C-shaped root canals is 0.9838 (CI 0.9775–0.9910), which is improved compared with the previous studies^38^. This paper found that adding an RFB module to a lightweight CNN can better learn and find tooth images’ depth features and details through multi-model training and ablation study. Experimental results prove that the effect of the RFB module simulating the human visual system to extract multi-scale features is very well. It helps significantly to reduce the number of parameters and time complexity. Initial efforts to identify and classify root canals were dominated by time-consuming and labor-intensive annotation by experienced dentists. Recently, breakthroughs have been made in the field of medical imaging using deep learning methods^39–44^. Most of the existing work on the classification of dental root canals mainly focuses on single canals, multiple canals, the binary of C-shaped root canals and multiple canals. According to the research investigation, two previous articles used deep learning to classify the root canals of mandibular first and second molars^38,45^. However, the results are not optimal, especially in classifying complex root canal types and a generalized approach does not exist. Meanwhile, previous studies did not consider the impact of model parameters and calculation size when classifying root canals. In most cases, limitations of hardware devices and usage environment will reduce the scope of their use scenarios. The proposed lightweight CNN model fills this deficiency and improves classification accuracy (Table 3).

In addition, to demonstrate the effectiveness of the network for learning C-shaped root canals, this paper implemented Score-CAM, an adaptive improvement of CAM^46^ that allows us to visualize the network’s concerns for different classifications. As shown in Fig. 9, the correct root canal region was focused by the decision-making network. Unfortunately, there are still many useless areas in the focus area of the visual map displayed by this network, which shows that the proposed network still needs improvement. This may also indicate that the lightweight shallow network is slightly lacking in the ability to extract more detailed features from tooth images.

Deep neural networks have become the most popular advanced technology in computer vision and medical imaging^47–49^. Neural networks are both computationally and memory intensive, making them difficult to deploy on embedded systems with limited hardware resources^50,51^. Previous studies on medical images have not considered this part, making it difficult to translate their results into actual clinical application. This will lead to wasted computing power and memory consumption of related devices. Therefore, when using the deep neural network method for medical image processing, it is necessary to consider the lightweight neural network model with the same accuracy. This reduces device resources and improves the conversion rate of research results to actual clinical applications.

This paper has several limitations. First, the training images are created by manual segmentation, which takes a lot of time. Therefore, an accurate automatic segmentation algorithm may be a further and valuable technical direction in tooth image. Second, the manually segmented tooth images have a complex background. The distance between teeth is very small, which inevitably blends with other tooth structures and the environment when segmenting the image, which to some extent prevents the model from learning the texture features of the area of interest. Third, translating the study results into practical clinical applications may still require a more significant data set to complete validation of the model’s generalization ability.

## Conclusion

In this work, a lightweight CNN model is proposed to detect the presence of C-shaped root canals in mandibular second molars. Compared with the methods of previous related studies, the proposed model has higher accuracy detecting C-shaped root canals and lower resource consumption. The proposed model improves feature extraction capability by incorporating an RFB module to enhance receptive fields. The RFB module which mimics the human eye’s receptive field, provides excellent help for feature extraction from lightweight CNN models and reduces the number of model parameters. The experimental results show that the proposed FARFB is better than that of dentists in detecting root canal types of mandibular second molars and better than other traditional methods in this work.

In future studies, we will use more clinical data for feedback training to improve the comprehensive efficiency of the model. Furthermore, we will implement the segmentation algorithm of the C-shaped root canal tooth image in the panorama based on the features extracted by the proposed method in this paper. Through rapid automated detection to replace cumbersome manual operations and then assist dentists in rapid clinical diagnosis.

## Data Availability

The raw/processed data required to reproduce these findings cannot be shared at this time as the data also forms part of an ongoing study. Further inquiries can be directed to the corresponding author.
